# Biomarkers of reproductive psychiatric disorders

**DOI:** 10.1192/bjp.2025.134

**Published:** 2025-06

**Authors:** Semra Etyemez, Kruti Mehta, Sonali Iyer, İpek Özdemir, Lauren M. Osborne

**Affiliations:** Department of Obstetrics & Gynecology, Weill Cornell Medical College, New York, NY, USA; Department of Psychiatry, Weill Cornell Medical College, New York, NY, USA; Russell H. Morgan Department of Radiology and Radiological Science, The Johns Hopkins University School of Medicine, Baltimore, MD, USA; Department of Biomedical Engineering, The Johns Hopkins University School of Medicine, Baltimore, MD, USA

**Keywords:** Biomarker, perinatal psychiatry, reproductive mental health, perinatal depression, postpartum depression

## Abstract

**Background:**

While biomarkers are widely used in other medical fields, psychiatry has yet to introduce reliable biological diagnostic tools. Female reproductive transitions provide a unique window of opportunity for investigating psychiatric biomarkers. Hormonal changes across menstruation, pregnancy, parturition and perimenopause can have dramatic effects on mental health in vulnerable individuals, enabling the identification of unique biomarkers associated with these fluctuations.

**Aims:**

This review integrates current evidence concerning potential biomarkers, with focus on recent human studies in perinatal depression, anxiety and obsessive–compulsive disorder, postpartum psychosis, premenstrual dysphoric disorder and perimenopausal depression.

**Method:**

We identified potential articles to be included in this narrative review by using PubMed to obtain articles in English since 2010 on the six conditions listed above, with the additional keywords of ‘biomarker’, ‘epigenetics’, ‘neuroactive steroid’, ‘immune’, ‘inflammatory’ and ‘neuroimaging’.

**Results:**

There is substantial published evidence regarding potential biomarkers of reproductive psychiatric disorders in the areas of epigenetics, neuroactive steroids, immune function and neuroimaging. This body of research holds significant potential to advance biomarker development, uncover disease mechanisms and improve diagnostic and therapeutic strategies, but there is as yet no clinically useful biomarker in commercial development for any reproductive psychiatric disorder.

**Conclusion:**

There is an urgent need for longitudinal, large-scale and multi-modal studies to examine potential biomarkers and better understand their functions across various stages of reproduction.

This past decade has witnessed intense effort to identify biomarkers that would significantly enhance the prevention, diagnosis and treatment of psychiatric disorders, ultimately leading us closer to ‘personalised medicine’. While biomarkers are widely used in other clinical disciplines, psychiatry has yet to develop reliable biological diagnostic and therapeutic tools. These challenges result from various factors including the heterogeneity of clinical presentations, high rates of comorbidity, pathophysiological complexity and individual variability in treatment responses.

The female reproductive transitions present a promising opportunity to study psychiatric biomarkers. The hormonal changes across menstruation, pregnancy, parturition and perimenopause can have substantial effects on the mental health of those at risk, and individual markers could emerge from our new understandings of these transitions. Illnesses at times of reproductive hormonal transition present a unique biological trigger that is not available in other psychiatric conditions. For these reasons, the search for biomarkers in conditions such as perinatal mood and anxiety disorders, premenstrual dysphoric disorder (PMDD) and perimenopausal mood disorders may also help in the search for biomarkers associated with psychiatric disease at other times, where the biological link is less obvious. Key factors under investigation include genetic, epigenetic, hormonal, immunological and neural circuit dynamics. (Of note, research in this area has focused largely on biological women, with little known about how either biology or symptom presentation may differ for those whose biological gender does not match their gender identity. This paper will use the terms appropriate to the population of each study; by and large, this will mean women.) This review summarises current knowledge on potential biomarkers and will focus on six conditions (though individual sections will discuss only those conditions for which we have evidence).

- Antenatal/perinatal/postpartum depression (AND/PND/PPD): there are many conflicting definitions of these terms. Some studies use them interchangeably, while others distinguish between antenatal (only during pregnancy), postpartum (only after delivery) and perinatal (both periods included). The DSM-5 definition is ‘with peripartum onset’, i.e., symptoms should have started either during pregnancy or within the first 4 weeks following delivery.^1^ These conflicting definitions are often inconsistent with both clinical experience (in which women can present with depression at any point in pregnancy or in the first postpartum year) and with biological evidence (particularly genetic evidence, which finds familial risk only for depression that begins within 6–8 weeks after childbirth).^2^ In this paper, we will use the term ‘antenatal’ when the data pertain specifically to the antenatal period, ‘postpartum’ when they pertain specifically to the postpartum period and ‘perinatal’ when the distinction is unclear or when the data span both periods.
- Perinatal anxiety: distinct from PND, characterised by heightened and often debilitating worry and anxiety during pregnancy and the postpartum period. While anxiety could also be subject to the same distinctions listed above for depression, most data span both pregnancy and postpartum and we will use ‘perinatal’ unless the data are exclusive to one period or the other.
- Perinatal obsessive–compulsive disorder (pOCD): onset or exacerbation of obsessive–compulsive disorder (OCD) symptoms during pregnancy or in the first year of postpartum. While OCD could also be subject to the same distinctions listed above for depression, most data span both pregnancy and postpartum and we will use ‘perinatal’ unless the data are exclusive to one period or the other.
- Postpartum psychosis (PPP): a rare but severe psychiatric emergency; an affective psychosis that occurs shortly after childbirth.
- PMDD: a severe form of premenstrual syndrome (PMS) occurring during the luteal phase of the menstrual cycle.
- Perimenopausal depression: a form of depression that presents in the perimenopausal period (the transitional years leading to menopause), characterised by the core symptoms of major depressive disorder (MDD), often accompanied by the physical and cognitive symptoms common to the menopausal transition. Other psychiatric illnesses can also be exacerbated in this period, but due to limited literature we will focus on depression only.

Early research on biomarkers of reproductive psychiatric illness focused on genetics, but this literature has yielded little conclusive information. Several twin and family studies show evidence for heritability in PPD, and candidate gene studies have focused on hormonal regulation (e.g. oestrogen receptors) and neurotransmitter systems (e.g. serotonin, dopamine), for example, polymorphisms in oestrogen receptor genes (ESR1 and ESR2) and the serotonin transporter gene.^2,3^ These studies tend to have positive findings for symptoms that arise shortly after childbirth and negative findings for symptoms arising at other times. In addition, a model using 116 gene transcripts, with a focus on oestrogen-responsive genes, predicted PPD with an accuracy of 88%.^4^ Similarly, genetic studies on PMDD indicate a potential hereditary component, with positive associations again with oestrogen and serotonin receptor candidate genes, although these findings lack consistent replication.^2^

More recently, biomarker research in this area has moved beyond genetics to explore blood-based epigenetic and protein markers as well as neuroimaging markers. While there are numerous interacting systems that could yield such markers, this paper will focus on those that have yielded the most comprehensive data thus far – epigenetic markers, neuroactive steroids (NASs), the immune system and inflammatory pathways and markers discernible through brain imaging. While the animal literature is rich, and we make reference to some fundamental older studies, we will primarily confine our remarks to human studies since 2010. Because this literature is vast, we make no claim to be comprehensive, but rather have chosen studies that we feel are most illuminating for our purposes. We have also focused on those studies that track endogenous biomarkers and have not considered studies of how these markers may change in response to treatment.

## Epigenetic markers

Epigenetic mechanisms are increasingly recognised as important to pathophysiology in both physical and mental disease. These mechanisms serve as dynamic and adaptive regulators of gene expression, and provide a real-life glimpse into how environmental elements shape health. Epigenetic mechanisms include DNA methylation, histone modification and chromatin remodelling, and epigenetic changes have been implicated in a wide range of diseases, from diabetes to autoimmune disease to cancer. Understanding such mechanisms can help us to unravel aetiology – and understanding how to reverse them, when maladaptive, can lead to novel drug discovery. Epigenetic mechanisms are increasingly being recognised as important contributors to the pathophysiology of PPD, although data on other reproductive psychiatric disorders is limited.

### AND

There is some evidence of epigenetic changes in AND. In a series of studies that began with animal research and extended into women, the Payne and Kaminsky group identified altered DNA methylation at two loci at the heterochromatin protein 1, binding protein 3 (HP1BP3 – associated with oestrogen signalling) and tetratricopeptide repeat domain 9B (TTC9B – associated with synaptic plasticity) as predictors of AND.^5,6^ Their study included blood samples from four prospectively collected cohorts, with DNA methylation data available from the first, second and/or third trimesters, depending on the cohort. Research has also linked epigenetic modifications in the nuclear receptor subfamily 3 group C member 1 (NR3C1 – associated with glucocorticoid signalling) gene to AND, with a positive correlation between increased methylation at CpG 4 and antenatal depressive symptoms.^7^ Another study found that DNA methylation changes in T-lymphocyte genes, particularly within a CD3 gene CpG cluster, were associated with AND.^8^

In addition, placental DNA methylation analysis identified 16 CpGs associated with AND, including two CpGs linked to the expression of ADAM23 (a disintegrin and metalloproteinase domain-containing protein 23) and CTDP1 (CTD phosphatase subunit 1) – genes implicated in neurodevelopment and neuropsychiatric disorders.^9^ These findings suggest that prenatal depression may induce epigenetic changes in the placenta, potentially influencing fetal brain development.

### PPD

A growing body of evidence suggests that DNA methylation variations can serve as predictive biomarkers of PPD. The Payne and Kaminsky biomarkers at TTC9B and HPIBP3, mentioned above under AND, were first studied for PPD. The group found that DNA methylation at two loci on these genes remains stable throughout pregnancy and significantly predicts PPD (area under the curve (AUC) 0.87).^10^ These initial studies were carried out in women who were euthymic in pregnancy and went on to develop PPD. Subsequent studies in independent cohorts provided further validation of the predictive value of these markers, and identified that the two genes in question were involved in oestrogen signalling (HP1BP3) and synaptic plasticity (TTC9B).^5,6^

Several studies have also examined epigenetic changes in the oxytocin receptor (OXTR, associated with oxytocin receptor) gene. Bell et al^11^ revealed an association between higher methylation levels at CpG site (−934) and increased PPD risk in women who were euthymic in pregnancy, particularly in those carrying the rs53576 GG genotype. Kimmel et al^12^ identified a significant association between PPD and an intronic region of OXTR located proximal to an oestrogen receptor binding region; CpGs in the region interacted with adverse life events to mediate the risk for PPD. Notably, adverse life events was associated with significantly increased DNA methylation in women who did not develop PPD.^12^ This finding was replicated in an independent cohort; moreover, DNA methylation in the region was negatively correlated with oestradiol (E2) ratios, and the interaction between E2 and OXTR methylation was positively associated with the ratio of allopregnanolone (ALLO) to progesterone (PROG). Interestingly, OXTR DNA methylation patterns have also been identified as potential epigenetic biomarkers of childhood adversity, with associations observed between PPD and mother–child bonding.^13^ Furthermore, lower OXTR methylation was associated with increased sensitivity to postnatal depressive symptoms in Latina mothers.^14^

Other studies identified additional epigenetic markers of PPD. One study identified positive correlation of NR3C1 methylation at CpG 2 with postpartum depressive symptoms.^7^ Another study investigated inflammatory markers of PPD and identified that lower TNF-α expression in late pregnancy was associated with PPD in patients who had high methylation at the FOXP3 (forkhead box P3) Treg-cell-specific demethylated region (TSDR). This suggests that discrimination-induced stress may enhance hypothalamic-pituitary-adrenal (HPA) activity, leading to immune suppression and altered inflammatory gene regulation.^15^

### PND

One study found that persistent depressive symptoms occurring during the perinatal period (either prenatally, postpartum or both) were associated with higher OXTR methylation within exon 3 and lower methylation in the intergenic region (IGR) between the oxytocin (OXT) and vasopressin (AVP) genes.^16^

### Perinatal anxiety

Two of the studies covered in the PPD section also investigated epigenetic changes in perinatal anxiety.^8,15^ One study observed that DNA methylation changes in T-lymphocyte genes, particularly within a CD3 gene CpG cluster, were associated with antenatal anxiety.^8^ The other study identified lower TNF-α expression in late pregnancy was associated with postpartum anxiety symptoms in those with elevated methylation at the FOXP3 TSDR.^15^

### pOCD

The Payne and Kaminsky group examined the epigenetic markers they identified for PPD in pOCD. They found that increased methylation at one of these same markers, TTC9B, along with increased methylation at the membrane-spanning 4A gene family (MS4A7), predicted OCD symptom exacerbation during pregnancy with moderate accuracy. Incorporating a methylome-wide approach, they further identified a DNA methylation proxy of the ratio of monocytes to non-monocytes, which improved the accuracy of the prediction model (AUC 0.75, 95% CI: 0.59–0.91). While promising, the small sample size (*N* = 24) and balanced case–control distribution may overestimate predictive performance. Further validation in larger, diverse cohorts is needed to confirm clinical applicability and explore whether these epigenetic changes are causally linked to pOCD.^17^

### PMDD

A study using a comparative whole-genome RNA-seq analysis between the mouse ventral hippocampus and lymphoblastoid cell lines from women with and without PMDD identified shared epigenetic biomarkers across species and cell types, including epigenetic modifiers of the ESC/E(Z) complex (ESC/E(Z):Extra Sex Combs/Enhancer of Zeste), a key regulator in the response to ovarian steroids.^18^

#### Summary

Overall, this latest research into epigenetic markers in reproductive psychiatric disorders highlights DNA methylation on hormone-responsive genes as potential biomarkers for reproductive psychiatric illness. Nevertheless, challenges still remain, particularly regarding tissue specificity, sample size limitations, disease specificity and the need for longitudinal validation. Subsequent studies must include multi-omics approaches to identify robust, reproducible epigenetic markers across diverse populations. Research is currently ongoing to examine these markers in PMDD and PPP. In addition, exploring tissue-specific DNA methylation differences – more specifically in brain tissue versus peripheral blood – will be critical in determining whether epigenetic changes are secondary to systemic inflammation or directly contribute to neuropsychiatric symptoms. Furthermore, given the role of oestrogen signalling in reproductive mood disorders, studying how hormonal fluctuations regulate such epigenetic signatures would provide valuable mechanistic insights. Integrating epigenetic findings with NAS and immune system data under a multi-omics framework may yield a more comprehensive understanding of reproductive psychiatric pathophysiology.

## Neuroactive steroids

NASs play a critical role in women’s health, with significant fluctuations throughout reproductive life stages. These naturally occurring metabolites of cholesterol are synthesised either in the brain or in the periphery (adrenal glands and ovaries). They act on the central nervous system by modulating behaviour and neurotransmitter function, increasing levels of serotonin and counteracting some effects of cortisol in the brain.^19^ NASs regulate the HPA axis and stress response.^20^ They act on several receptors, primarily gamma-aminobutyric acid (GABA)-A,^; 19,20^ the receptor for the primary inhibitory neurotransmitter in the central nervous system. Research highlights the roles of various NASs, such as pregnanolone, dehydroepiandrosterone (DHEA) oestradiol (E2) and testosterone, in mood and anxiety symptoms related to reproductive transitions, with a significant focus on allopregnanolone (ALLO), a metabolite of progesterone (PROG).^21^ ALLO and pregnanolone are positive allosteric modulators of the GABA-A receptor, producing anxiolytic and sedative effects, while their isomers, isoallopregnanolone and epipregnanolone, act as negative modulators, reducing GABAergic neurotransmission.^22^

NAS synthesis varies during key reproductive stages – menstruation, the perinatal period and perimenopause. PROG and ALLO exhibit low levels during the follicular phase, increase throughout the luteal phase and then decline rapidly in the late luteal phase before menstruation.^23,24^ In the perinatal period PROG and ALLO rise steadily to supraphysiological levels during pregnancy and drop sharply after delivery.^21^ The highly variable transition to menopause includes increased E2^25^ and decreased PROG in the luteal phase^25^ and fluctuations in ALLO.^24^ Psychiatric conditions during these reproductive transitions are linked to changes in NASs. While there is some limited literature on the roles of other NASs – such as pregnanolone, allotetrahydroDOC, DHEA, DHEA-S and testosterone – in mood and anxiety disorders related to reproductive transitions,^24^ the majority of the evidence currently available focuses on the metabolites of PROG, and we have therefore focused this section on those molecules. In perimenopausal depression, we also briefly consider the role of changes in E2, as there is a more substantial literature in this area.

### AND

A study of 96 third-trimester pregnant women found that those with elevated depression scores (Montgomery–Åsberg Depression Rating Scale – self-rating version; MADRS-S ≥ 13) had significantly lower ALLO levels and a negative correlation with concurrent self-reported depression scores,^26^ while another study of 14 women linked lower PROG and combined ALLO and pregnanolone levels in the second trimester to increased negative emotional reactions to stress.^27^

Our group found no significant association between concurrent depressive symptoms and PROG and ALLO levels during pregnancy in women with a prior mood disorder (*n* = 60).^28^ Similarly, in another study (*n* = 111) we found no significant relationship between ALLO levels and depression during pregnancy.^29^ Another study in 284 pregnant women found no relationship between ALLO and concurrent mood symptoms during pregnancy,^30^ but did find that those who were homozygous for the minor allele in a polymorphism on the AKR1C2 gene (the gene for a key enzyme in the PROG metabolic pathway) had lower levels of ALLO and a steeper rise in depressive symptoms across pregnancy.

### PPD

While at least one older study found no significant differences in postpartum ALLO levels between women with and without PPD at 9 weeks or 6 months postpartum,^31^ our group identified both cross-sectional and predictive relationships between NASs and postpartum depressive symptoms. We reported that higher ALLO levels at week 6 postpartum were associated with worse mood symptoms^29^ and found that lower ALLO levels in euthymic women during the second trimester predicted postpartum-onset PPD, with each additional ng/mL decreasing the odds of developing PPD by 63%.^28^ Similarly, a subsequent study found that participants with a one-unit increase in PROG as well as in the isoallopregnanolone/pregnanolone ratio at the third trimester (T3) had higher odds of developing PPD, while those with a one-unit increase in the log pregnanolone/PROG ratio had lower odds.^32^ Conversely, another found no differences in either pregnancy or postpartum levels of pregnanolone, ALLO, pregneolone or their ratios to PROG between those with and without PPD.^33^ Recent clinical trials with synthetic ALLO also support a role for GABAergic NASs in PPD.^34^ These findings suggest that it may not be the absolute levels of NASs that matter, but rather the dynamics of their withdrawal postpartum and changes in GABA-A receptor sensitivity, which may contribute to psychiatric symptoms. This underscores the importance of precise timing in evaluating potential biomarkers.

### PND

Another study of 75 women found that those at risk for depression during pregnancy or the postpartum (The Edinburgh Postnatal Depression Scale (EPDS) >10 or a history of PND or MDD) had elevated levels of 5-alpha-dihydroprogesterone (5α-DHP) and 5-beta-dihydroprogesterone (5β-DHP) (the intermediary molecules between PROG and, respectively, ALLO and pregnanolone) and ALLO compared to healthy controls,^35^ while the same group showed that at-risk women also had higher PROG and pregnanolone levels, with pregnanolone positively associated with Hamilton Rating Scale for Depression (HAMD-17) and Hamilton Anxiety Rating Scale (HAM-A) scores, and lower GABA levels negatively associated with these scores.^35^

In light of this inconclusive evidence in AND, PPD and PND, some researchers have also explored ratios of NASs to their precursors in the PROG metabolic pathway. The Deligiannidis group^35^ found significant differences in the ratio of ALLO to 5α-DHP among participants with PND, those at risk and healthy controls, and in the ratio of pregnanolone to 5β-DHP between healthy controls and at-risk women, although no differences were noted between healthy controls and those with PND. In addition, another study reported a correlation between increased ratios of ALLO to PROG and pregnanolone to PROG with concurrent depressive symptoms during the first and second trimesters compared to healthy controls in a diverse population.^36^

A recent meta-analysis of 13 studies (2509 women, including 849 with peripartum depressive symptoms (PDSs), encompassing several of the studies listed above) examined the relationship between ALLO blood concentrations and PDSs. Overall, no significant differences in ALLO levels were observed between women with and without PDSs at any time point during the second or third trimesters or postpartum (*p* > 0.05). However, subgroup analyses revealed higher ALLO levels in women with PDSs at gestational weeks 21–24 and 25–28. Methodological factors, including sample type and analytical techniques, influenced the results. These findings underscore the challenges posed by heterogeneous time points, cohorts and methods in understanding the role of NASs like ALLO in PDSs.^37^

### Perinatal anxiety

Our group found a correlation between low ALLO levels in the second trimester and subsequent postpartum anxiety,^38^ while higher ALLO levels were associated with increased concurrent anxiety symptoms at 6 weeks postpartum.^29^ Our recent study broadened this scope to explore the entire PROG metabolic pathway in women with and without clinically significant anxiety, excluding those with comorbid depression. We found a significant moderating effect of anxiety group on the relationship between PROG and ALLO. In particular, when compared to healthy controls, women with anxiety exhibited a more pronounced shift from the third trimester to postpartum week 6 in metabolism towards the end-points of the PROG metabolic pathway.^39^ We also found that women with at least one minor allele at AKR1C2 rs1937863 (the gene for a key enzyme in the PROG metabolic pathway) had higher concentrations of 5α-DHP and a lower ratio of ALLO:5α-DHP at T3.

The variability in these results may stem from differences in study designs, including how anxiety is defined and timed, the presence of comorbid psychiatric disorders and the methods used for quantifying NASs.

### PMDD

There is a substantial body of literature on fluctuations in NASs throughout the menstrual cycle and their role in the symptoms of PMDD. The primary NASs whose fluctuations have been studied during the menstrual cycle and are relevant to PMDD include PROG, its metabolite ALLO and E2. Ovarian steroid hormone levels may not differ between women with PMDD versus without;^40^ rather, symptom onset in PMDD may be connected with an abnormal response caused by altered sensitivity of central nervous system receptors to normal hormonal fluctuations.^41^ Researchers have proposed the Dimensional Affective Sensitivity to Hormones across the Menstrual Cycle (DASH-MC) framework, suggesting that ALLO mediates hormonal effects on mood. This model links atypical responses to E2 and PROG fluctuations with distinct affective changes, offering a transdiagnostic approach to hormone sensitivity in psychiatric disorders.^42^ Some evidence shows that PMDD symptoms start with the increase in PROG and E2 levels during the luteal phase, worsen in the luteal phase as hormone levels decline and improve with the start of menstruation.^43^ A recent study did report, however, that PROG had a distinct pattern in PMDD, featuring an early leftward shift that begins in the periovulatory phase followed by a rapid decrease during the mid to late luteal phase.^44^ Other studies lend support to a difference in hormone levels. Elevated ALLO levels and ALLO/PROG ratios in women with PMDD compared to those without PMDD have also been observed, with a curious inverse relationship between ALLO levels and symptom severity.^45^ Another study found that isoallopregnanolone potentially alleviates negative mood symptoms by antagonising ALLO’s effects on the GABA-A receptor.^46^ Brain activity in emotion-processing regions during the late luteal phase has been shown to correlate with the isoallopregnanolone/ALLO ratio in PMDD, while the opposite was observed in controls.^47^

Furthermore, PMDD patients show altered sensitivity to ALLO across the menstrual cycle, with changes in saccadic eye velocity (SEV).^48^ This study aimed to examine how PMDD patients respond to intravenous ALLO across the menstrual cycle, using SEV as a measure of GABA-A receptor activity. PMDD patients exhibited a decreased SEV response in the follicular phase compared to the luteal phase, while controls showed the opposite pattern. These findings suggest that PMDD patients have altered sensitivity to ALLO, which is influenced by the menstrual cycle.^48^

There is also evidence of disrupted ALLO–GABA-A receptor interactions, contributing to PMDD symptoms and increased stress sensitivity, particularly because of dysregulation of the HPA axis during the luteal phase.^49^ A randomised study showed that blocking 5-alpha reductase (which converts PROG to ALLO) reduced PMDD symptoms, supporting the idea that NAS regulation is involved in PMDD.^50^

PMDD also involves disrupted autonomic nervous system regulation, with lower heart rate variability (HRV) and altered stress responses.^51^ Baseline ALLO levels significantly predicted peak high frequency of the heart rate (HF-HRV) changes in the PMDD group, suggesting its role in modulating stress responses in PMDD.^51^ Another study investigated the relationship between neuroactive PROG metabolites, such as ALLO, and HPA axis function in women with PMDD. While there were no differences in diurnal cortisol secretion or dexamethasone suppression between PMDD patients and healthy controls, higher ALLO levels in PMDD patients were associated with blunted nocturnal cortisol levels compared to controls with lower ALLO. This suggests that diurnal cortisol secretion in PMDD may be influenced by luteal phase ALLO levels, potentially explaining inconsistencies in previous studies of HPA axis function in PMDD.^52^

The studies on PMDD inevitably point toward the contribution of fluctuations of the levels of such hormones, including PROG and its metabolite ALLO, to causation and exacerbation of PMDD. The key findings point towards the altered responsiveness of the central nervous system toward such fluctuations of the hormones, and not toward abnormalities of the levels of the hormones, and this may be contributing to the severity of the symptoms, more significantly in the luteal phase. Also, impaired interaction of ALLO with the GABA-A receptor, increased sensitivity to stress and dysfunction of the autonomic nervous system, more significantly in the luteal phase, are contributing factors to PMDD’s pathogenesis, and targets of treatment could be modulation of NAS action and the HPA axis.

### Perimenopausal depression

As women transition to menopause, ovarian function gradually declines, particularly marked by decreased E2 and PROG levels. This weakens these hormones’ negative feedback on the pituitary gland, leading to increased follicle-stimulating hormone (FSH) and luteinising hormone.^53^ E2 fluctuations are linked to significant changes in FSH, resulting in more menstrual cycles without follicular development and reduced PROG levels.^54^ Changes in PROG and its metabolites during the perimenopausal period are believed to contribute to mood fluctuations, with studies linking NAS variability to depression, anxiety and irritability.^55,56^ and mixed evidence on whether differences in absolute levels are important.^54,57,58^ There is also considerable evidence concerning changes in E2 at the menopausal transition, with studies indicating correlation between higher depressive symptoms or mood fluctuations and increased E2 variability^54,59^ or a sudden drop in E2 levels.^60,61^

#### Summary

In summary, NASs are crucial for mood regulation, with levels of PROG and its metabolites in particular fluctuating across reproductive stages. While NAS dysregulation is implicated in perinatal affective disorders, PMDD and perimenopausal mood changes, inconsistencies in research findings highlight the need to focus on the dynamics of NAS fluctuations and their impact on GABA-A receptor function, rather than absolute levels. Future research should prioritise longitudinal studies with frequent NAS sampling, careful attention to timing of symptom onset, investigation of subunit-specific GABA-A receptor modulation and exploration of interactions with other neurotransmitter systems and the HPA axis. Standardised protocols and clinical trials are crucial for clarifying the role of NASs in these disorders and developing targeted interventions to improve women’s mental health.

## Immunological biomarkers

The immune system has a major influence on homeostasis in the brain and mood modulation through different cytokines, chemokines and other immunological mechanisms. Recent research has revealed that inflammation and immune signalling can directly affect neurotransmitter systems, neuroplasticity and synaptic strength, and hence brain dysfunction, and a solid body of evidence links increased inflammation to depression outside of reproductive transitions.^62^

Among these key immunological players, cytokines and chemokines represent a group of small signalling proteins produced by immune cells and have a central function in cell communication during immune responses such as inflammation.^63^ These findings hint at a possible role of immune markers in regulating mood during the menopausal transition, but the nature of this relationship is not fully understood. Elevated inflammatory markers, such as interleukin-6 (IL-6), may contribute to neuroinflammation, further disrupting neurotransmitter pathways and possibly affecting the HPA axis function that regulates the response to stress and mood. Further, more research is needed to determine whether active immune dysregulation is a causal risk factor in mood disturbances or merely a measure of the biological response to the hormonal changes.^64^ In addition to cytokine signalling, certain psychiatric disorders may be associated with the peripheral release of extracellular vesicles, including exosomes and microvesicles, which influences intercellular communication. Extracellular vesicles contain bioactive molecules such as cytokines, microRNAs, proteins and lipids, which influence the function and behaviour of recipient cells. In psychiatric disorders, extracellular vesicles were reported to be involved in both neuroinflammation and neurodegeneration by transporting pro-inflammatory factors and microRNAs through the blood brain barrier (BBB), subsequently affecting synaptic connectivity in the brain immune subset.

Another very important immune-modulating pathway involved in psychiatric disorders is the kynurenine (KYN) pathway. Tryptophan (Trp) is either metabolised to serotonin or down the KYN pathway into neuroprotective or neurotoxic metabolites. In an inflammatory environment, inflammatory cytokines such as IFN-γ, interleukin-1β (IL-1β) and tumour necrosis factor-alpha (TNF-α) stimulate indoleamine 2,3-dioxygenase (IDO), which degrades Trp towards the KYN metabolites, thus reducing serotonin levels and potentially causing depressive symptoms.^65^ The KYN can be further converted into the neurotoxic quinolinic acid (QUIN), which activates *N*-methyl-D-aspartate (NMDA) receptors, or into the neuroprotective kynurenic acid (KYNA), an NMDA antagonist with anti-inflammatory effects.^66^ Dysregulation of this pathway, influenced by inflammatory cytokines, has been implicated in depression, anxiety and psychosis, particularly during the perinatal period in women, suggesting that these metabolites along the KYN pathway may be candidates for biomarker studies of reproductive psychiatric disorders.^67^

The following sections will delve into the specific immunological biomarkers identified in each of these disorders, summarising key research studies that have contributed to our current understanding.

### AND

Biancardi et al^68^ found higher levels of IL-6 and TNF-α in depressed women with a history of trauma during the second trimester compared to depressed women without a trauma history, suggesting that trauma prior to the pregnancy may worsen immune dsyregulation and be related to depressive symptoms during pregnancy. In contrast, another study illustrated the opposite correlation between increased levels of TNF-α and depressive symptoms in the third trimester, indicating dynamic immune changes during pregnancy and in patients with AND.^69^ In addition, Leff Gelman et al^70^ reported positive correlations between depressive symptoms and cytokines such as interleukin 2 (IL-2), IL-6, TNF-α, Th2-related mediators interleukin 13 (IL-13), interleukin 10 (IL-10) and interleukin 9 (IL-9) and the T-helper 17 (Th17)-associated cytokine interleukin 17A (IL-17A) during the third trimester. Furthermore, a study by Miller et al^71^ reported that higher levels of pro-inflammatory cytokines such as IL-1β, interleukin 23 (IL-23) and interleukin 33 (IL-33) in cerebrospinal fluid (CSF) were correlated with an increased risk for AND, suggesting that these inflammatory markers in the brain may play a more significant role in AND than peripheral inflammation alone. Interestingly, this study found little correlation between levels in CSF and those in the periphery. Most other studies have focused on the periphery alone, and it is unknown how correlations found in the periphery – even when associated with illness – may be correlated with action in the central nervous system.

Besides cytokine studies, several studies have shown that the KYN pathway is also influenced by inflammatory activity in AND. They found that prenatal Trp levels and the KYN/Trp ratio moderate the association between IL-6 levels and AND. Moreover, levels of IL-6, QUIN, TNF and KYN in the second trimester showed strong individual accuracy in predicting depression risk and severity in the third trimester, making them potential biomarkers for AND.^72^ In addition, a longitudinal study by Sha et al found that elevated levels of inflammatory cytokines (IL-6 and TNFα) and alterations in markers of Trp metabolism (QUIN and KYN) during pregnancy can accurately predict depressive symptoms during pregnancy.^73^ Another recent study by the same group reported that low expression of critical KYN pathway enzymes such as quinolinate phosphoribosyltransferase (QPRT) and 2-amino-3-carboxymuconate-6-semialdehyde decarboxylase (ACMSD) in placenta samples was associated with increased levels of plasma cytokines and a dysregulated KYN metabolite pattern, which were linked to depressive symptoms, suggesting the role of inflammation and KYN pathway enzymes as possible therapeutic targets in peripartum depression.^74^ In addition, some of the studies have shown that elevated C-reactive protein (CRP) levels, alongside lower Trp levels, were correlated with AND.^75,76^ In contrast, women with depressive symptoms in the second trimester showed lower CRP levels.^77^

### PPD

Recent research has identified key immunological biomarkers associated with PPD, particularly focusing on cytokines, the KYN pathway and extracellular vesicles.^78,79^ As reviewed by Osborne and Monk,^67^ numerous studies have found elevated pro-inflammatory markers such as IL-6 and TNF-α associated with increased risk of PPD, while a few studies have reported decreased cytokine levels linked with PPD symptoms, suggesting that cytokine–cortisol interactions may vary among individuals with PPD.^80^ In addition, one study reported that prenatal Trp levels and the KYN/Trp ratio moderated the association between IL-6 levels and PPD.^72^ Two studies have shown that low levels of anti-inflammatory cytokines, particularly IL-10, increase susceptibility to PPD. This highlights the protective role of anti-inflammatory states against mood disturbances.^81,82^ Some researchers have attempted to consolidate such findings by developing composite inflammation scores; these have been able to predict the onset of PPD, but less effectively than a clinical history of prior depression.^81^ The same research team conducted metabolic profiling studies, revealing variations in immune-related metabolic pathways associated with PPD. They identified distinct metabolic profiles connected to postpartum depressive symptoms, indicating that disruptions in lipid metabolism and immune-related metabolites may play a key role in the development of PPD.^83^ Furthermore, another study by Min et al^84^ found a positive correlation between elevated Th17 cells and related cytokine IL-17A with both PPD and anxiety, which further supports the role of Th17-mediated inflammation in these conditions. Our own group has explored the connection between cellular immune dysfunction and PPD. We found that defects in regulatory T-cells are linked to immune dysregulation and contribute to PPD,^85^ and that altered mRNA communication in extracellular vesicles during pregnancy, tied to reduced autophagy, is also associated with subsequent PPD, suggesting that molecular signalling pathways involving immune cells may further contribute to the disorder.^86^

Research on the KYN pathway has also provided insights into underlying pathways of PPD. Sha et al^87^ have shown that hormonal changes, especially higher E2 levels, were linked to a pro-inflammatory profile and neurotoxic KYN metabolites, while PROG was associated with an anti-inflammatory profile, shedding light on the hormonal and immune interactions influencing PPD. In addition, another study has explored the role of the KYN pathway and kynurenic aminotransferase (KAT) alleles in PPD following caesarean section in Chinese women; their findings indicate that postpartum depressive symptoms (PDSs) are associated with decreased KAT activity and an increased QUIN/KYNA ratio, although no significant relationship was found between KAT alleles and PDSs.^88^

In summary, the current literature emphasises the complex role of cytokine profiles, immune cell activity, metabolic pathways and molecular signalling in the etiology and progression of PPD. These findings suggest that immune-related biomarkers are likely to be the key to the identification of women at risk of PPD and may provide new targets for treatment. However, methodological variations and small sample sizes are notable across these studies, with effect sizes differing depending on the demographic characteristics and immune markers measured.

### PND

In our own study, we examined the role of pro-inflammatory cytokines in depression across the perinatal period and found increased levels of IL-6 and macrophage inflammatory protein-1 α, chemokine (C-C motif) ligand 3 (MIP-1α/CCL3) beginning in the third trimester and continuing through the early postpartum in more depressed women. This study also found that women with higher pro-inflammatory cytokine values were more likely to be Hispanic, but less likely to be African American or overweight/obese compared to women with lower pro-inflammatory cytokine values, suggesting differential patterns in cytokine levels by race, ethnicity and overweight/obesity status.^89^

Small sample sizes, heterogeneity of mood assessment tools and cytokine detection techniques in these studies affect generalisability and consistency across findings. These findings highlight how inflammatory changes during pregnancy may contribute to PND, with immune dysregulation potentially persisting into the postpartum period. Understanding the trajectory of these immune alterations is of key importance to the discovery of biological mechanisms influencing mood disturbance during the perinatal phase.

### Perinatal anxiety

Several studies have reported positive associations between high levels of pro-inflammatory cytokines such as IL-6, C-X-C motif chemokine ligand 8 (IL-8/CXCL-8) and TNF-α and/or a composite pro-inflammatory score with higher anxiety symptoms during pregnancy and postpartum.^89,90^ Notably, Sherer et al^91^ found a negative correlation, where lower levels of pro-inflammatory cytokines were associated with higher anxiety. This finding is particularly relevant as it examines anxiety independently of depression, suggesting that there may be distinct immune mechanisms underlying perinatal anxiety versus comorbid anxiety and depression. In addition, population differences may be important to these differential findings.^92^ Furthermore, some studies have found that a pro-inflammatory profile is accompanied by elevated levels of anti-inflammatory cytokines (IL-13, IL-10), possibly as a compensatory response,^93^ whereas other studies have linked increased levels of inflammation with increased cortisol concentrations in participants with higher levels of anxiety and depressive symptoms.^94^ Notably worthy of mention is that only a few studies have been conducted in populations with anxiety alone, without comorbid depression – so findings may be partially explained by that discrepancy.

In addition, studies exploring chemokine profiles in perinatal anxiety have revealed elevated levels of pro-inflammatory chemokines, such as IL-8/CXCL-8, monocyte chemoattractant protein 1 chemokine (C-C motif) ligand 2 (MCP-1)/CCL2 and macrophage inflammatory protein 1 beta chemokine (C-C motif) ligand 4 (MIP-1β)/CCL4, as well as an increase in composite pro-inflammatory chemokine score in women with moderate to severe anxiety, suggesting that chemokine profiles may play a crucial role in immune dysregulation and mood disturbances,^95^ although some studies have not found differences.^91^ We conducted a study in perinatal anxiety without comorbid depression in Pakistan and showed a distinct pattern of cytokine and chemokine profiles associated with perinatal anxiety, differing from those found in other populations, indicating that population-level factors may influence immune responses.^96^ Specifically, elevated chemokine activity across pregnancy in highly anxious women was observed. Our group has also developed a composite factor score by performing principal component analysis (PCA) and clustering innate immune markers as well as chemokine markers with anxiety symptoms in a longitudinal framework, enabling us to assess immune patterns across multiple time points in this population.^96^ Few studies in perinatal anxiety have moved beyond cytokines and chemokines to consider dysregulation in immune cells; one such study found that increases in the cytotoxic to helper T-cell ratio and altered balance between Th17 and Treg cells were associated with perinatal anxiety symptoms.^91^

### PPP

Research suggests that immune dysregulation, particularly alterations in cytokine levels and Trp metabolism, plays a vital role in the development of PPP.^97^ Bergink et al^98^ found distinct immune changes in first-onset PPP, including elevated MCP-1/CCL2 and reduced anti-inflammatory glucocorticoid receptor alpha (GR-α) compared to healthy postpartum and non-postpartum controls. In addition, higher IL-1β levels were observed in healthy postpartum women, indicating immune shifts specific to the postpartum period that may contribute to PPP’s pathophysiology. Furthermore, they reported significantly elevated T-cells (both T-effector and T-regulatory cells) in healthy women postpartum, whereas patients with PPP lacked this elevation. Instead, they had increased monocyte levels and upregulated immune-related monocyte genes compared to both postpartum and non-postpartum controls. Their findings suggest a balanced interplay between pro-inflammatory and anti-inflammatory responses, indicating that the immune system adapts during the postpartum period to maintain equilibrium and prevent excessive inflammation.^98^ Similarly, Sathyanarayanan et al^99^ also showed elevated IL-6 and uniquely elevated IL-8/CXCL-8 in the PPP group, suggesting a specific role of IL-8/CXCL-8 in PPP development. In addition, studies on Trp metabolism found imbalances in neurotoxic and neuroprotective metabolites, with increases in 3-hydroxykynurenine (3HK) and decreases in KYNA, suggesting immune activation as a potential etiology for neuropsychiatric symptoms in PPP.^100^

### PMDD

Recent evidence suggests that the inflammatory response, which may be triggered by hormonal fluctuations, could contribute to the mood disturbances, fatigue and physical symptoms commonly seen in PMDD and PMS.^101^ Barone et al^102^ presented elevated levels of the pro-inflammatory markers IL-8/CXCL-8 and TNF-α during the luteal phase in a PMDD group compared to controls. Also, they found positive association between premenstrual symptom severity with higher levels of IL-8/CXCL-8, highlighting the potential role of inflammation in the manifestation of PMDD symptoms. Despite all these results, evidence in this area is still limited, and further studies are needed to better understand the underlying immunological mechanisms of PMDD.

### Perimenopausal depression

Perimenopausal depression is a significant psychiatric illness that arises in the context of the menopausal transition, characterised by mood fluctuations, anxiety and cognitive symptoms. Growing evidence suggests that hormonal disturbances, neuroinflammation and oxidative stress contribute to its development.^103^ One of the earliest immunological changes observed in perimenopausal depression is an increase in pro-inflammatory cytokines. As reviewed by Liang et al,^103^ there have been many studies reporting elevated levels of interleukin-8 (IL-8), TNF-α, IL-1β and IL-6 in women experiencing perimenopausal depression. These cytokines are important mediators of neuroinflammation, synaptic dysfunction and neuronal apoptosis, all contributing to mood disorders.^103^ In addition, Matthews et al^104^ also suggested that perimenopausal women with higher depressive symptoms exhibit increased fibrinogen and plasminogen activator inhibitor type 1 (PAI-1), both of which are markers of systemic inflammation and hypercoagulability. These findings highlight an increased cardiovascular risk associated with perimenopausal depression, further linking immune dysregulation to broader health concerns. Furthermore, decreased E2 levels favour a pro-inflammatory state, which in turn enhances IDO activity, leading to a shunt of Trp metabolism through the KYN pathway. This yields an overproduction of QUIN, a neurotoxic metabolite, that hyperstimulates NMDA receptors, resulting in glutamatergic excitotoxicity, oxidative stress and neurodegeneration. Concurrently, the levels of KYNA, a neuroprotective metabolite, are reduced, further destabilising the neuroprotection versus neurotoxicity balance, potentially leading to mood disturbances, cognitive impairment and increased vulnerability to depression during perimenopausal transition.^103^ Due to this strong link among immune dysregulation, KYN metabolism and neuroinflammation, targeting these pathways could provide novel therapeutic opportunities for the prevention and treatment of perimenopausal depression.

#### Summary

In summary, the studies reviewed here strongly support the role of differential patterns of immunological biomarkers in reproductive psychiatric disorders. Among all mentioned disorders, there is convincing evidence of emerging biomarkers including cytokines, extracellular vesicles and the KYN pathway metabolites. Methodological inconsistency, small sample size and variation in the design of studies restrict generalisation. Large-scale longitudinal studies with multi-omics approaches that track sequential changes in immune biomarkers should be the top priority in future research to clearly establish the bidirectional relationship among hormonal fluctuations, immune dysregulation and symptoms. Research in relatively unexplored conditions, such as pOCD, will also be critical for bridging the gaps. The advancement of clinical application of immunological biomarkers opens new perspectives for personalised treatment that can help improve the outcomes of women’s mental health across life stages.

## Neuroimaging biomarkers

Research into epigenetic, endocrinological and immunological markers of psychiatric illness in women is expanding, but studies examining the underlying neurocircuitry remain limited. Understanding normal physiological variations in the central nervous system throughout the female lifespan is important for elucidating the development of psychiatric illness in women. While specific imaging biomarkers have not yet been characterised, the field is promising, and the current literature provides valuable guidance for future studies. Given the unique challenges associated with imaging studies in pregnant individuals, it may take time before clinically predictive biomarkers are identified. However, characterising the neuroimaging features of these conditions may be the first step in the development of biomarkers. This section describes the imaging modalities used in studying peripartum depression, anxiety, psychosis, PMDD and perimenopause, along with recent key findings.

### AND, PND and PPD

Imaging studies in pregnant women are limited, with most data focusing on PPD. Recent research suggests that pregnancy induces significant, lasting changes in brain structure, function and neural plasticity, which are generally considered adaptive to the reproductive transition.^105^

There are no studies focusing exclusively on the antenatal period. For PPD, recent structural neuroimaging studies report abnormal changes in grey matter volume, cortical thickness, white matter integrity and network connectivity. A comparison of 29 women with PPD and 23 healthy postpartum women found a correlation between EPDS scores and cortical and subcortical differences in PPD, with greater cortical thickness in frontal and visual areas.^106^ Conversely, decreased cortical thickness in women with PPD in the right inferior parietal lobule was reported in another study comparing 21 PPD women to 18 controls.^107^ Additionally, increased grey matter volume in the left dorsolateral prefrontal cortex (DLPFCL) and the right anterior insula and increased surface area in several regions, including the DLPFC and insula, was found in women with postpartum depressive symptoms versus healthy controls.^107,108^ Recent studies have shifted focus from individual brain regions to examining the combined regions of the default mode network (DMN) and reward network. A few studies have used diffusion tensor imaging (DTI) techniques in PPD and have reported altered white matter integrity of the cortical thalamic circuits in PPD women compared to healthy controls.^109,110^

While imaging is largely confined to postpartum, there are some studies that conduct imaging postpartum but consider depressive symptoms during both pregnancy and postpartum. One recent study found that the severity of both AND and PPD symptoms, along with antepartum ALLO concentrations, predicted volume changes in brain regions within the DMN and reward network involved in emotion regulation and cognition among postpartum women.^111^

While research on structural neuroimaging is limited, functional magnetic resonance imaging (fMRI) has extensively identified brain regions linked to PPD symptoms. There are no studies confined to the antenatal period. Deligiannidis et al^33,112^ found altered functional connectivity within areas of the DMN and in corticocortical and corticolimbic regions in PPD compared to healthy controls. In addition, they noted a relationship between postpartum ALLO concentrations and resting-state functional connectivity (RSFC) in the DMN for PPD.^112^ The DMN, which is involved in self-referential thinking and emotional processing, is particularly significant in PPD as disruptions in this network may contribute to the negative self-appraisal and rumination often observed in the disorder. Other fMRI studies observed reduced amygdala response to negative stimuli and decreased striatal activation after reward in PPD.^113,114^ The reward network is also critical in PPD, as altered responses to reward may be associated with the anhedonia often seen in depression. Additional studies noted alterations within the DMN, limbic system (LIN) and interhemispheric connectivity.^115^ By contrast, Schnakenberg et al^116^ did not find any structural or functional changes in PPD.

Structural and functional imaging can identify brain regions associated with symptoms, while magnetic resonance spectroscopy (MRS) and positron emission tomography (PET) imaging can examine differences of biochemical systems at the molecular level in the brain. An initial MRS study of PPD found no significant differences in occipital cortex GABA levels compared with healthy controls.^31^ Another study reported higher glutamate concentrations in the medial prefrontal cortex (MPFC).^117^ Conversely, lower combined glutamate plus glutamine and combined *N*-acetylaspartate (NAA) plus *N*-acetylaspartylglutamate were reported in the left DLPFC, with no differences in the anterior cingulate gyrus (ACG), in PPD versus healthy controls.^118^ In addition, blunted HPA axis responses were found to be linked with low levels of glutamate and glutamine in the ACG in PPD.^119^ A combined fMRI and MRS study noted a link between cortical GABA concentration and RSFC in the DMPFC in postpartum women, with no GABA differences, in PPD and healthy controls.^112^ PET studies in PPD revealed elevated brain monoamine oxidase-A (MAO-A) binding in the ACC and prefrontal cortex,^120,121^ and decreased postsynaptic serotonin 1 A receptor binding in regions of the ACC, the mesiotemporal and the lateral orbitofrontal cortices,^122^ while no differences were found in dopaminergic D2/3 receptor binding in the striatum.^123^

### Perinatal anxiety

Very few papers have examined imaging features in women with perinatal anxiety without mood symptoms. Those that exist are confined to the postpartum period. Two studies reported that greater neural responses to negative infant cues were correlated with higher postpartum anxiety symptoms.^124,125^ Increased maternal anxiety was associated with stronger connections between caregiving behaviours and amygdala and right posterior superior temporal sulcus connectivity.^126^ In addition, several studies have explored perinatal anxiety in relation to PPD. Women with PPD with anxiety showed changes in the subgenual anterior cingulate cortex (sgACC) and dynamic functional connectivity (dFC) between the sgACC and superior temporal sulcus compared to women with PPD and without anxiety.^127^ Functional connectivity density linked both anxiety and depression to common and specific circuit disruptions.^128^ PPD with anxiety disrupted the lateral prefrontal-striatal circuit, while PPD without anxiety affected the occipito-parieto-frontal circuit.^128^ Irregularities in the DMN distinguished PPD from PPD with anxiety.^129^

### pOCD

One study that compared postpartum women with OCD to those without (as well as to a comparison group of mothers past the postpartum period) found that, compared to healthy postpartum women, those with OCD had heightened stress responses (both self-report and endocrine) in response to psychosocial stress, and that this response was associated with a distinct pattern of activation in the orbitofrontal and temporal cortices.^130^

### PPP

Research on neuroimaging in PPP is limited, but some evidence of structural and functional differences has been noted. One study found decreased volume in the parahippocampal gyrus, anterior cingulate cortex (ACC), postcentral gyrus and superior temporal gyrus (STG) in postpartum women with recent psychotic episodes compared to at-risk women without such episodes.^131^ Two other studies reported increased connectivity between the DLPFC and other areas along with functional differences in working memory and emotional recognition in women at risk of PPP.^132^ In addition, one study found that, while connectivity in areas related to executive functioning increased overall, it decreased during emotional tasks.^133^

### PMDD

A major recent advancement in the structural neuroimaging literature on PMDD comes from the Comasco lab, which conducted studies based on an unprecedentedly large sample. The findings highlight the value of large-scale, phase-specific imaging studies to better characterise the neurobiological basis of PMDD. These studies investigated structural changes in grey matter in women with PMDD during the luteal phase. One study found higher depressive symptoms in PMDD correlated with lower bilateral amygdala volume.^134^ Another study reported that PMDD is associated with greater white matter volume in the right uncinate fasciculus and increased fractional anisotropy in several brain regions compared to controls.^135^ The same group also investigated structural differences in PMDD in both mid-follicular and late luteal phases and found that women with PMDD consistently showed thinner cortices and reduced gyrification compared to controls, regardless of menstrual cycle phase – suggesting trait-like brain differences.^136^ In addition, menstrual cycle-related changes in cortical architecture were observed across all participants.^136^ While Dubol et al^137^ reported reduced volume in the cerebellum, right amygdala and ventral putamen, earlier studies noted increased volume in posterior cerebellar volume and in the left hippocampal gyrus^138^ and decreased parahippocampal gyrus in PMDD compared to healthy controls.^139^

fMRI studies reveal significant changes in brain reactivity to emotional stimuli during the luteal phase in PMDD. These include increased amygdala response decreased prefrontal cortex activity to negative and social stimuli^140,141^ and reduced ACC activity to social and emotion regulation tasks.^142,143^ In addition, increased insula activation occurs during emotional tasks and social tasks,^142,144^ alongside hyperconnectivity in the basal ganglia and thalamus.^145^

PET and MRS imaging studies have examined biochemical changes in PMDD. Increased glucose metabolism in the cerebellum and abnormal activation during working memory tasks in the DLPFC were observed from the follicular to the late luteal phase in PMDD compared to healthy controls.^146,147^ Alterations in the serotonergic system included a smaller increase in serotonin 1A receptor (5-HT_1A_) binding in dorsal raphe nuclei from the follicular to the late luteal phase in PMDD,^148^ altered levels of serotonin precursor 5-hydroxytryptophan (5-HTP) in prefrontal regions and the striatum correlated with symptom severity in PMDD.^149^ Recently, increased availability of serotonin transporter in the midbrain during the luteal phase was described.^150^ MRS studies found higher GABA levels in the occipital cortex from the follicular to the mid- and late luteal phases^151^ and lower GABA levels in the ACC/MPFC and left basal ganglia.^152^ However, some studies reported no differences in glutamate or other metabolite levels in the MPFC and cingulate gyrus between PMDD and healthy controls.^153,154^

### Perimenopausal depression

To date, brain imaging research has not focused on perimenopausal depression. (While there are reports of brain changes in response to the hormone shifts that are characteristic of perimenopause – with possible implications that could be important for future studies on depression – those studies are outside the scope of this paper. Similarly, there are imaging studies of cognitive changes in perimenopause as well.^155^) Wang et al^156^ is the only study to have used DTI to investigate this subgroup and found alterations in white matter microstructure in the insula and subcortical areas that correlated with depressive symptoms in perimenopausal women.

#### Summary

In summary, most neuroimaging studies to date on psychiatric illness at times of reproductive hormonal transition has focused on PPD and PMDD. Structural imaging studies have shown changes in grey matter volume, cortical thickness and white matter integrity, with significant findings in the DMN and reward networks linked to emotion regulation. Functional imaging, most notably fMRI, has highlighted abnormal connectivity in regions such as the amygdala, prefrontal cortex and insula during emotional stimuli. MRS and PET imaging have detected biochemical changes in neurotransmitter systems, such as glutamate, GABA, serotonin and MAO-A. However, heterogeneity in imaging modalities, methodologies and study designs, as well as small sample sizes, present challenges for validating and replicating findings. Longitudinal, multi-modal imaging studies are required to confirm previous findings, identify reliable biomarkers and explore their potential to predict future illness. Although MRI can be conducted during pregnancy and postpartum period, logistical challenges such as participant availability, motion artefacts and minimising stress or discomfort complicate study designs and data collection. The use of PET imaging is further restricted during pregnancy because of radiation exposure, thereby underscoring the need for alternative approaches. Nonetheless, PMDD and perimenopause offer the unique window of opportunity to study neurobiological changes across reproductive transitions. These studies can develop our understanding of psychiatric illness at other stages of reproductive hormonal transition, such as pregnancy and postpartum; improve diagnostic and treatment strategies; and address ethical and practical challenges in vulnerable populations.

## Synthesis and future directions

**Fig. 1:**
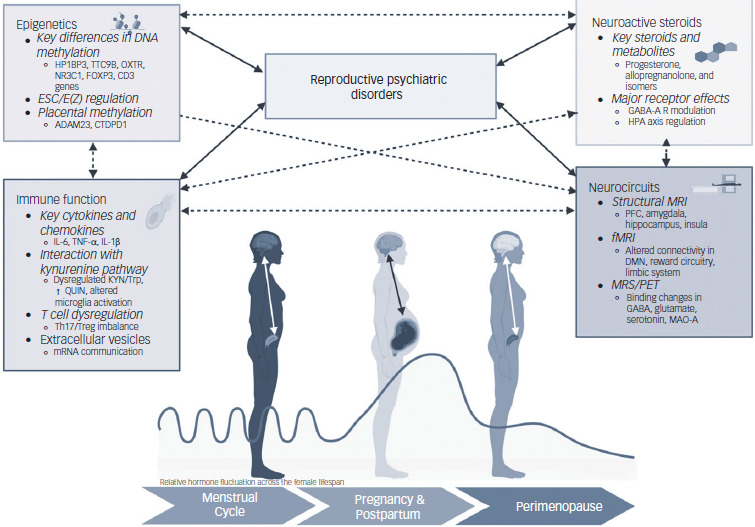
Potential biomarkers of reproductive psychiatric disorders (created with BioRender.com). HP1BP3, heterochromatin protein 1, binding protein 3; TTC9B, tetratricopeptide repeat domain 9B; OXTR, oxytocin receptor gene; NR3C1, nuclear receptor subfamily 3 group C member 1; FOXP3, Forkhead box P3; ESC/E(Z), extra sex combs/enhancer of zeste; ADAM23, a disintegrin and metalloproteinase domain-containing protein 23; CTDP1, CTD phosphatase subunit 1; IL-6, interleukin-6; TNF-α, tumour necrosis factor-alpha; IL-1β, interleukin-1β; KYN, kynurenine; Trp, tryptophan; QUIN, quinolinic acid; Th17, T-helper 17; GABA, gamma-aminobutyric acid; HPA, hypothalamic–pituitary–adrenal; MRI, magnetic resonance imaging; PFC, prefrontal cortex; fMRI, functional magnetic resonance imaging; DMN, default mode network; MRS, magnetic resonance spectroscopy; PET, positron emission tomography; MAO-A, monoamine oxidase-A.

Biomarker research in reproductive psychiatric disorders, including exploration of epigenetics, immunological markers, NASs and neuroimaging, holds immense promise for enhancing our understanding of these conditions (Table 1 and Fig. 1). While PPD has been the primary focus of research, a comprehensive understanding of reproductive psychiatric disorders requires integrating findings across all reproductive transitions (including AND and PND, perinatal anxiety, pOCD, PPP, PMDD and perimenopausal depression) and biomarker types (epigenetics, immunology, NASs, neuroimaging). The state of the literature thus far is primarily one of association, not causation – so few of the markers covered here are close to being studied as clinically useful tests. There are nevertheless some commonalities that can point us toward future research.

**Table 1:** Summary of potential biomarkers

Type of illness	Epigenetics	Neuroactive steroids	Immune function	Neuroimaging
Antenatal depression	↑ HP1BP3 and TTC9B methylation if euthymic earlier in pregnancy, ↓ if depressed earlier in pregnancy ↑ NR3C1 methylation signalling ↓ ADAM23 methylation ↓ CTDP1 methylation	Conflicting data regarding ALLO, PROG levels	↑ Pro-inflammatory IL-1β, IL-23, IL-33 in CSF ↑ Peripheral IL-6, QUIN, TNF and KYN T2 predicted T3 risk ↑ Peripheral IL-2, IL-6, IL-13, IL-10, IL-9 and IL-17A T3 in severe depression and anxiety Conflicting evidence regarding TNF-α and CRP levels	Severity of AND symptoms, along with ↑antepartum ALLO concentrations, predicted volume changes in brain regions within the DMN and reward network
Postpartum depression	↑ HP1BP3 and TTC9B methylation if euthymic in pregnancy, ↓ if depressed in pregnancy Conflicting evidence, ↑ and ↓ OXTR methylation ↑ FOXP3 TSDR methylation	Conflicting data regarding ratios of different NASs Conflicting data regarding ALLO – although some evidence of ↓ ALLO levels at T2 predicting PPD ↑ Progesterone and ↑ negative:positive modulator ratios at T3 predict PPD	↑ IL-6 and TNF-α ↓ Anti-inflammatory cytokines such as IL-10 ↑ Th17 cells and IL-17A ↑ E2 levels associated with pro-inflammatory profile; ↑progesterone associated with anti-inflammatory profile ↓ KAT activity, ↑ QUIN/KYNA ratio	↑ Cortical thickness in frontal and visual areas, ↓ cortical thickness right inferior parietal lobule ↑ Grey matter volume in DLPFCL and right anterior insula PPD severity and ↑ ALLO predict volume changes within DMN Altered white matter integrity of cortical thalamic circuits fMRI: altered functional connectivity in corticocortical and corticolimbic regions, ↓ amygdala response, ↓ striatal activation MRS and PET: mixed data on glutamate uptake; link between ↑ cortical GABA and resting-state functional connectivity in DMPFC; ↑ MAO-A binding in ACC and prefrontal cortex; ↓ HT-1A receptor binding in ACC, mesiotemporal and lateral orbitofrontal cortices
Perinatal depression	↑ OXTR methylation ↓ IGR methylation	↑ 5α-DHP, 5β-DHP	↑ IL-6 and CCL3 beginning T3 and continuing early postpartum Differential patterns in cytokine levels by race, ethnicity and overweight/obesity status	
Perinatal anxiety	↑ CD3 methylation lymphocytes ↑ FOXP3 TSDR methylation	↓ ALLO levelsT2 ↑ ALLO levels 6 weeks postpartum Larger shift from T3 to postpartum to end-points in PROG metabolic pathway	Conflicting evidence (↑ and ↓) pro-inflammatory cytokines IL-6, IL-8/CXCL-8 and TNF-α, depending on population Conflicting evidence on chemokines IL-8/CXC L8, MCP-1/CCL2 and MIP-1β/CCL4 Increase in the cytotoxic to helper T-cell ratio and altered shifts in the balance between Th17 and Treg cells	Changes in functional connectivity between sgACC and superior temporal sulcus PPD with anxiety disrupts lateral prefrontal-striatal circuit Stronger connections among anxiety, amygdala and right posterior superior temporal sulcus connectivity Links between postpartum anxiety and altered maternal brain responses to infant cues
Perinatal obsessive-compulsive disorder	↑ Methylation of MS4A7 and TTC9B associated with obsessions			Distinct pattern of activation in the orbitofrontal and temporal cortices
Postpartum psychosis			↑ MCP-1/CCL2, IL-6, IL-8/CXCL-8 ↓ GR-α and IL-1β ↓ T-effector and T-regulatory cell levels ↑ 3HK and KYNA – suggests involvement with tryptophan metabolism	↓ Volume of parahippocampal gyrus, anterior cingulate cortex, postcentral gyrus and superior temporal gyrus ↑ Connectivity between DLPFC and other areas
Premenstrual dysphoric disorder (PMDD)	Modifiers of ESC/E(Z) complex expression	Early leftward shift of PROG in periovulatory phase with rapid ↓ in mid/late luteal phase 5α-reductase blocker led to decrease in PMDD symptoms ↑ ALLO levels and ALLO/PROG ratios	↑ IL-8/CXCL-8 and TNF-α during luteal phase Symptom severity positively correlated with IL-8/CXCL-8	sMRI: - ↑ and ↓ cerebellar volume (conflicting data) ↑ Volume in left hippocampal gyrus ↓ In parahippocampal gyrus, right amygdala, ventral putamen and lingual, fusiform and inferior occipital areas ↓ Volume in bilateral amygdala and right uncinate fasciculus ↓ In cortical thickness fMRI: luteal phase, ↑ amygdala and insula response, ↓ PFC and ACC activity, ↑ increased insula activation, hyperconnectivity in basal ganglia and thalamus MRS and PET: ↑ glucose metabolism in cerebellum; smaller ↑ in 5-HT_1A_ receptor binding in dorsal raphe nuclei; altered 5-HTP levels in prefrontal areas and striatum from late luteal to mid follicular phase. Conflicting data regarding GABA uptake
Perimenopausal depression	–	↑ E2 variability positively correlates with depressive symptoms Significant ↓ E2 and PROG associated with depressive symptoms	↑ IL-8, TNF-α, IL-1β and IL-6 ↑ Fibrinogen and PAI-1 associated with ↑ depressive symptoms ↓ E2 favours pro-inflammatory state → Altered kynurenine pathway	Alterations in white matter microstructure in insula and subcortical areas

3HK, 3-hydroxykynurenine; 5α-DHP, 5-alpha-dihydroprogesterone, intermediary molecule between PROG and ALLO; 5β-DHP, 5-beta-dihydroprogesterone, intermediary molecule between PROG and pregnanolone; 5-HT1A, serotonin 1A receptor; ACC, anterior cingulate cortex; ADAM23, a disintegrin and metalloproteinase domain-containing protein 23; ALLO, allopregnanolone, metabolite of PROG; AND, antenatal depression; CCL2, chemokine (C-C motif) ligand 2; CCL-3, chemokine (C-C motif) ligand 3; CCL4, chemokine (C-C motif) ligand 4; CRP, C-reactive protein; CSF, cerebrospinal fluid; CTDP1, CTD phosphatase subunit 1; CXCL-8, C-X-C motif chemokine ligand 8; DLPFCL, Dorsolateral prefrontal cortex; DMN, default mode network; E2, oestradiol; FOXP3, Forkhead box P3; GR-α, glucocorticoid receptor alpha; HP1BP3, heterochromatin protein 1, binding protein 3; HT-1A, serotonin 1A; IL-1β, interleukin 1-Beta; IL-6, interleukin 6; IL-10, interleukin 10; IL-17A, interleukin 17A; IL-23, interleukin 23; IL-33, interleukin 33; KAT, kynurenic aminotransferase; KynA, kynurenic acid; KYN, kynurenine, tryptophan metabolite; MAO-A, monoamine oxidase-A; MCP-1, monocyte chemoattractant protein 1; MIP-1β, macrophage inflammatory protein 1 beta; MPFC, medial prefrontal cortex; MRS, magnetic resonance spectroscopy; MS4A7, membrane-spanning 4A subfamily member 7; NAS, neuroactive steroids; NR3C1, nuclear receptor subfamily 3 group C member 1; OXTR, oxytocin receptor gene; PAI-1, plasminogen activator type 1; PET, positron emission tomography; PFC, prefrontal cortex; PND, perinatal depression; PPD, postpartum depression; PROG, progesterone; QUIN, quinolinic acid; sgACC, subgenual anterior cingulate cortex; T2, second trimester; T3, third trimester; Th17, T-helper 17; TNF, tumour necrosisf factor; TSDR, treg-cell-specific demethylated region; TTC9B, tetratricopeptide repeat domain 9B.

Much of the data – no matter which section we fit it into above – points to individual vulnerability to hormone fluctuation. Epigenetic markers are on hormone-responsive genes; immune markers vary as hormones change; and genetic polymorphisms that underlie key steps in the metabolism of hormones appear to be related to symptoms. It is thus clear that our next step is to determine not which of the various protein markers examined here is the ’smoking gun’ that can serve as a predictive biomarker of all reproductive psychiatric illness, but rather whether one or a few of them they can be used as markers for the various reasons for individual vulnerability to hormonal change. Some individuals, for example, may be vulnerable because of genetic defects in genes for enzymes; others may be vulnerable because of unique patterns of interaction between the immune system and neurotransmitters in the setting of hormone change; still others because of failures in receptor plasticity in response to hormone change. Useful biomarkers will be those that capture a shared biological signal, and can indicate risk in those who may be vulnerable for different reasons.

To arrive at such biomarkers, future research must prioritise longitudinal studies with standardised methodologies and diverse samples to address inconsistencies and limitations in current findings. Critically, exploring the complex interplay among genetic, epigenetic, hormonal, immunological and neural factors is essential, utilising a multi-omics approach. While some studies examine the interplay between hormones and brain imaging (such as those of the Deligiannidis group),^112,157^ surprisingly few studies look at interactions among these systems, which is a clear necessity to advance this research further.

It will also be crucial to delineate whether these illnesses are distinct entities or variations of common psychiatric disorders, especially concerning timing of onset, and identify individuals sensitive to hormonal fluctuations. Paying attention to timing and predictive utility is also of high importance. Biomarkers will be most helpful if they can predict who will become ill – and therefore studies that collapse together periods that may have distinct biological risks (such as pregnancy and postpartum) will continue to be of limited use. Ultimately, translating these findings into clinical practice, through improved diagnostics, risk prediction and personalised treatments, is the ultimate goal, requiring collaborative, interdisciplinary efforts.

## Data Availability

Data availability is not applicable to this article as no new data were created or analysed in this study.
